# A New Proof of Concept in Bacterial Reduction: Antimicrobial Action of Violet-Blue Light (405 nm) in *Ex Vivo* Stored Plasma

**DOI:** 10.1155/2016/2920514

**Published:** 2016-09-28

**Authors:** Michelle Maclean, John G. Anderson, Scott J. MacGregor, Tracy White, Chintamani D. Atreya

**Affiliations:** ^1^The Robertson Trust Laboratory for Electronic Sterilisation Technologies (ROLEST), Department of Electronic and Electrical Engineering, University of Strathclyde, 204 George Street, Glasgow, UK; ^2^Department of Biomedical Engineering, University of Strathclyde, Wolfson Centre, 106 Rottenrow, Glasgow, UK; ^3^Office of Blood Research and Review, Center for Biologics Evaluation and Research, Food and Drug Administration, Bethesda, MD, USA

## Abstract

Bacterial contamination of injectable stored biological fluids such as blood plasma and platelet concentrates preserved in plasma at room temperature is a major health risk. Current pathogen reduction technologies (PRT) rely on the use of chemicals and/or ultraviolet light, which affects product quality and can be associated with adverse events in recipients. 405 nm violet-blue light is antibacterial without the use of photosensitizers and can be applied at levels safe for human exposure, making it of potential interest for decontamination of biological fluids such as plasma. As a pilot study to test whether 405 nm light is capable of inactivating bacteria in biological fluids, rabbit plasma and human plasma were seeded with bacteria and treated with a 405 nm light emitting diode (LED) exposure system (patent pending). Inactivation was achieved in all tested samples, ranging from low volumes to prebagged plasma. 99.9% reduction of low density bacterial populations (≤10^3^ CFU mL^−1^), selected to represent typical “natural” contamination levels, was achieved using doses of 144 Jcm^−2^. The penetrability of 405 nm light, permitting decontamination of prebagged plasma, and the nonrequirement for photosensitizing agents provide a new proof of concept in bacterial reduction in biological fluids, especially injectable fluids relevant to transfusion medicine.

## 1. Introduction

Bacterial contamination of* ex vivo* stored injectable biological fluids such as blood and blood components preserved in plasma is a major complication for transfusion medicine, resulting in both wasteful discarding of valuable blood products and, more significantly, health risks for recipients of contaminated donor blood [1, 2]. Major progress has been made in the provision of a safe supply of blood components, and measures such as more effective donor screening, extensive laboratory testing protocols, and the application of bacterial reduction methods have significantly reduced the risk of transfusion-transmitted bacterial infections [1–3]. Nevertheless, the risk of bacterial transmission has not been completely eliminated and there is a recognised need for continued research to improve the efficacy of these methods and to minimise incidental adverse changes in biological fluids, such as cellular blood components preserved in plasma, which can compromise product quality and safety [4–6].

A number of bacterial reduction methods have been developed for plasma treatment, and pathogen reduced plasma is routinely used [7], with several of these methods now licensed for use in North America and Europe [5]. The original methods developed for plasma treatment included the use of solvent/detergent and methylene blue in combination with visible light [8–11]. More recently, developed methods have employed ultraviolet (UV) light. Exposure to amotosalen (S-59) plus long-wave ultraviolet (UVA) light [12, 13] and treatment with riboflavin and UV light [7, 14] have been developed to treat both plasma and platelets. Whilst light-based processes have typically used photosensitive chemicals to generate microbicidal effects, a UV-C-based pathogen reduction system without a requirement for photoactive substances has been developed and is undergoing clinical efficacy and safety testing [15–17].

It is generally accepted that all these methods have limitations [5, 7], and because the full extent of future microbiological challenges cannot be predicted, pathogen reduction technologies will remain an active area of investigation in transfusion medicine well into the future [1, 4].

Here, we report the first proof-of-concept results on the use of a novel visible violet-blue light method that does not require the addition of photosensitive chemicals for inactivation of bacterial pathogens in plasma. This method utilises light with a peak wavelength of 405 nm, which causes photoexcitation of endogenous microbial porphyrin molecules and oxidative damage through reactive oxygen species [18]. 405 nm light has previously been shown to inactivate a wide range of bacterial pathogens in laboratory tests [19–28] as well as in hospital settings with use as an environmental disinfection technology [29–31] and also potential for wound decontamination applications in clinical settings [32–34]. An advantage of this technology over UV light for certain applications is that, even at irradiance values and dose levels that are bactericidal, it can be applied safely for human exposure. Therefore, we envisioned that this feature makes 405 nm light of potential interest for decontamination of injectable stored biological fluids such as blood plasma or plasma containing cellular blood components. Tests on bacterial-seeded plasma were carried out on both small-scale liquid samples and artificially contaminated prebagged plasma. Direct treatment of prebagged plasma was facilitated by the highly transmissible properties of 405 nm light, and the bacterial inactivation results obtained using this novel approach are described for the first time in this paper.

## 2. Materials and Methods

### 2.1. Bacterial Cultures

The organisms used in this study were* Staphylococcus aureus* NCTC 4135,* Staphylococcus epidermidis* NCTC 11964, and* Escherichia coli* NCTC 9001. Cultures were obtained from the National Collection of Type Cultures (NCTC), Colindale, UK. For experimental use, bacteria were cultured in 100 mL nutrient broth at 37°C under rotary conditions (120 rpm) for 18 h. Broths were centrifuged at 3939 ×g for 10 minutes and the pellet was resuspended in 100 mL phosphate buffered saline (PBS) and serially diluted to obtain the required cell density (colony-forming units per millilitre, CFU mL^−1^) for experimental use. All culture media were sourced from Oxoid Ltd. (UK).

### 2.2. Plasma

Lyophilised rabbit plasma (LRP020, E&O Laboratories, UK) was reconstituted using sterile distilled water. Fresh frozen human plasma (approximately 300 mL bag volume) was obtained from the Scottish National Blood Transfusion Service (SNBTS, UK) and defrosted before experimental use. Study involving human subjects protocol was approved by FDA Risk Involved in Human Subjects Committee (RIHSC, Exemption Approval # 11-036B) and by the University of Strathclyde Ethics Committee (letter dated 10 February 2011). Rabbit plasma and human plasma suspensions were seeded with known concentrations of bacterial contaminants by adding bacterial-PBS suspension to the plasma.

### 2.3. 405-nm Light Source

The 405 nm light sources used were rectangular arrays of 99 LEDs in an 11 × 9 matrix (Opto Diode Corp., USA). The array had a centre wavelength close to 405 nm, with a bandwidth of approximately 10 nm at full width at half maximum (FWHM). The LED array was powered by a direct current supply, and, for thermal management, the LED array was bonded to a heat sink and fan, thus ensuring that heating had no effect on the test samples exposed to the 405 nm light (device patent pending [35]).

### 2.4. 405 nm Antimicrobial Light Treatment

Three arrangements were employed for exposure of three different sample volumes: 3 mL, 30 mL, and approximately 300 mL (whole plasma transfusion bags). For exposure of 3 mL sample volumes, the samples were held in the well of a 12-well microplate (without the lid), and the LED array was mounted in a polyvinyl chloride (PVC) housing which positioned the array approx. 3 cm directly above the sample. Irradiance at the sample surface was measured to be approximately 100 mWcm^−2^ (measured by using a radiant power meter and photodiode detector; LOT-Oriel Ltd.).

For exposure of 30 mL sample volumes, the human plasma was held in a sterile 90 mm Petri dish with the lid on. The LED array was positioned 8 cm directly above the closed sample dish, providing irradiance of approximately 8 mWcm^−2^, through the lid, at the centre of the sample dish.

For exposure of plasma bags, a test rig was constructed which held two 405 nm LED arrays at a distance of 12 cm above the horizontally positioned plasma bag. This arrangement provided irradiance of approximately 5 mWcm^−2^ at the centre position of the plasma bag, taking into account a 20% reduction in irradiance as the light transmits through the bag layer. In order to investigate the influence of higher irradiance on bacterial inactivation, plasma bags were also exposed using irradiance of 16 and 48 mWcm^−2^. This higher irradiance was achieved by using two high-power 405 nm LED arrays (PhotonStar Technology, UK), with 14 nm FWHM.

All experimental systems were held in a shaking incubator (72 rpm; 25°C) to allow continuous sample agitation and maintain exposure conditions. Samples seeded with bacterial contamination were treated with increasing exposures of 405 nm light. Control samples were held in identical conditions but shielded from the 405 nm light.

The optical profiles of the light distribution across the Petri dishes and transfusion bags (plotted using MATLAB R2012b software) demonstrate the nonuniform irradiance of the plasma (Figures 2(a) and 3(a)); however, continuous agitation of the plasma samples during treatment ensures uniform mixing of the plasma contaminants. Negligible variation was recorded across the 22 mm diameter of the 3 mL samples.

### 2.5. Determination Whether Light Induces Toxicity within Human Plasma

To ensure that bacterial inactivation was not the result of the plasma becoming toxic upon exposure to 405 nm light,* S. aureus* (1 × 10^3^ CFU mL^−1^) was seeded into 3 mL plasma that had been preexposed to 1.08 kJcm^−2^ 405 nm light at irradiance of 100 mWcm^−2^ (the highest dose employed in the present study) and samples were taken at 30 min intervals for up to 3 hr.

### 2.6. Bacterial Enumeration

Following 405 nm light exposure, samples were either plated onto nutrient agar using an automatic spiral plater (Don Whitley Scientific, UK) or manually spread by using sterile L-shaped spreaders, depending on the expected population density of the samples. Sample plates were incubated at 37°C for 24 hours and then enumerated with the surviving bacterial load reported as colony-forming units per millilitre (CFU mL^−1^).

### 2.7. Inactivation Data Analysis

Results are reported as surviving bacterial load (log_10_ CFU mL^−1^) as a function of dose and are presented as mean values from triplicate independent experiments (*n* = 3). Dose (J cm^−2^) is calculated as the product of the irradiance (W cm^−2^) multiplied by the exposure time (sec), with the irradiance value being the maximum measured at the centre position of the sample dish/bag. Significant differences in the results were identified using 95% confidence intervals and one-way analysis of variance (ANOVA) with Minitab software Release 16. For dose response curves the dose that reduces log_10_ CFU count at 0 dose by 50% was estimated. This 50% log_10_ reduction was estimated using curve fitting software (GraphPad Prism V6) and quadratic or 5PL variable slope sigmoidal curves with R-squared fits in excess of 90%.

### 2.8. Optical Analysis of Plasma

The transmission values for rabbit plasma and human plasma, PBS, and the blood bag material were measured by using a BioMate 5 UV-Visible Spectrophotometer (Thermo Spectronic). Analysis was carried out in the wavelength range of 220–700 nm. Fluorescence spectrophotometry (RF-5301 PC spectrofluorophotometre; Shimadzu, US) was used to determine whether plasma or PBS contained photosensitive components which could be excited by 405 nm light. Excitation was carried out at 405 nm and emission spectra were recorded between 500 and 700 nm.

## 3. Results

### 3.1. Inactivation of Microbial Contaminants in 3 mL PBS and Plasma

**Figure 1:**
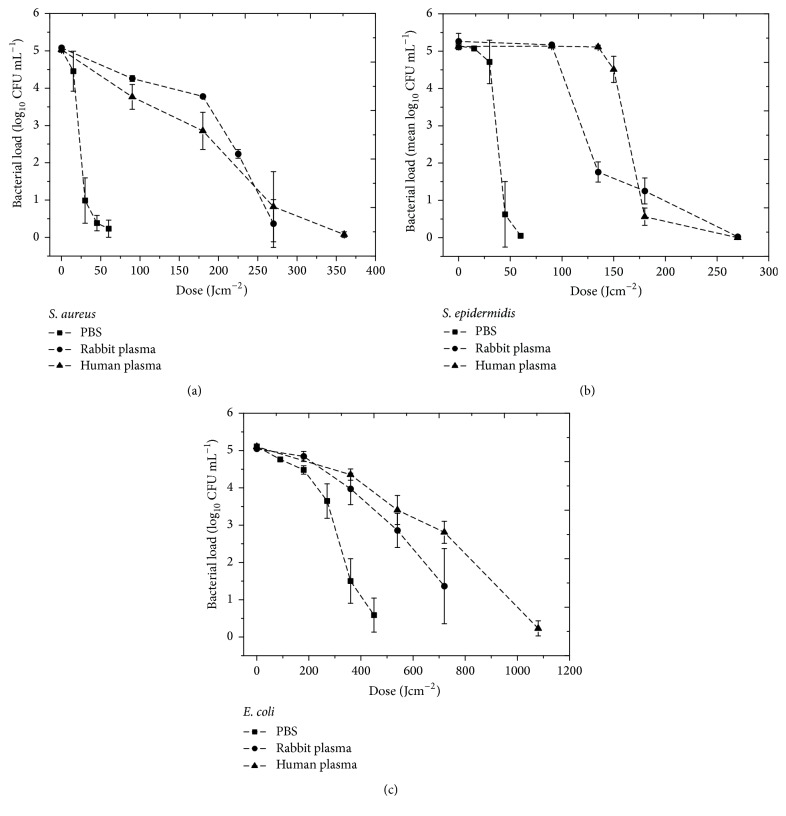
Inactivation of bacterial contamination: (a)* S. aureus*, (b)* S. epidermidis*, and (c)* E. coli*, in phosphate buffered saline (PBS) rabbit plasma and human plasma by exposure to 405 nm light with irradiance of approximately 100 mWcm^−2^ (*n *= 3 ± SD). Nonexposed control samples for all experiments demonstrated no significant change in population over the exposure period [*P* ≥ 0.05].

Results from the exposure of PBS, rabbit plasma, and human plasma seeded with bacterial contamination (10^5^ CFU mL^−1^) to 100 mWcm^−2^ 405 nm light are presented in Figure 1. Results demonstrate that bacterial inactivation in PBS is achieved using the lowest dose. Data for* S. aureus* (Figure 1(a)) show that near complete inactivation (<10 CFU mL^−1^ surviving) of the organism in PBS was achieved after exposure to a dose of 60 Jcm^−2^. To achieve a comparable reduction in rabbit plasma and human plasma, 4.5 times the dose was required (270 Jcm^−2^ compared to 60 Jcm^−2^). 50% log_10_ reductions were estimated to occur at doses of 23, 224, and 181 Jcm^−2^ for PBS, rabbit plasma, and human plasma, respectively.

Similar inactivation kinetics was observed for* S. epidermidis* (Figure 1(b)), although this species showed comparatively greater susceptibility to 405 nm light when exposed in plasma. The 50% log_10_ reductions were obtained in PBS, rabbit plasma, and human plasma at 36, 121, and 174 Jcm^−2^ respectively. Reduction of* E. coli* contamination required markedly increased doses (Figure 1(c)). The 50% log_10_ reductions required doses of 328, 585, and 742 Jcm^−2^ for PBS, rabbit serum, and human serum, respectively. For inactivation in PBS, 450 Jcm^−2^ was required for near complete inactivation (<10 CFU mL^−1^ surviving): 7.5 times more than observed with the staphylococci. Inactivation of* E. coli* contamination in plasma again required increased doses compared to suspension in PBS, with a 5-log_10_ reduction in human plasma achieved after a dose of 1.08 kJcm^−2^.

### 3.2. Determination of Light Induced Toxicity within Human Plasma

No significant change in the seeded 10^3^ CFU mL^−1^ population [*P* = 0.663] was evident in the bacterial contamination added to plasma after exposure, thus indicating no residual toxicity in 405 nm light-exposed plasma which could induce the inactivation of microbial contaminants.

### 3.3. Inactivation of Contaminants in Larger Volumes of Human Plasma

#### 3.3.1. 30 mL Volume in Covered Sample Dish

**Figure 2:**
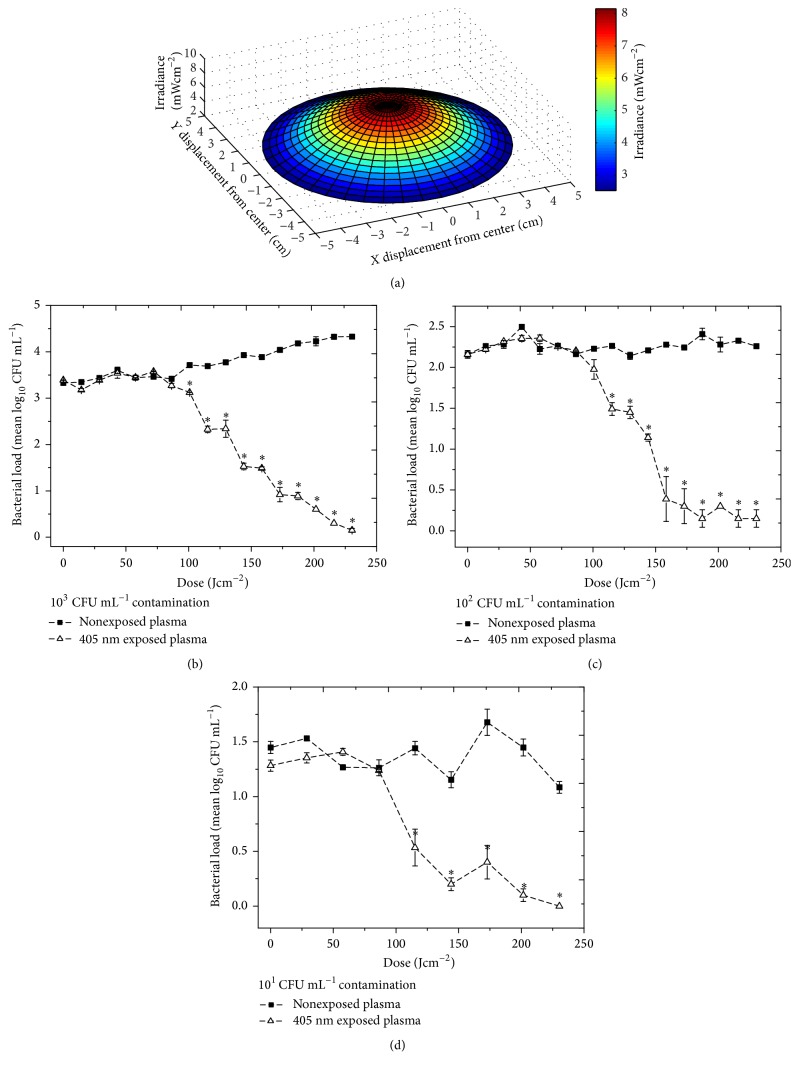
Inactivation of* S. aureus* contamination in 30 mL volumes of human plasma held in a closed sample dish by exposure to 405 nm light. (a) Three-dimensional model demonstrating the irradiance profile across the sample dish, with irradiance of ~8 mWcm^−2^ at the centre. Populations of (b) 10^3^, (c) 10^2^, and (d) 10^1^ CFU mL^−1^ were used as the seeding densities (*n *= 3 ± SE). Asterisks (*∗*) represent data points, where the bacterial levels in light-exposed plasma were significantly different from the equivalent nonexposed control [*P* ≤ 0.05].

Figure 2 demonstrates the inactivation of low density* S. aureus* contamination in 30 mL plasma in a closed Petri dish using irradiance of ~8 mWcm^−2^. Results for a seeding density of 10^3^ CFU mL^−1^ (Figure 2(b)) demonstrate that exposure to doses of greater than 100.8 Jcm^−2^ caused significant inactivation of the contamination [*P* = 0.030], with near complete inactivation achieved with 230.4 Jcm^−2^. Control contamination levels rose significantly by approximately 1-log_10_ over the course of the experiment [*P* < 0.001]. Similar results were observed for inactivation of the 10^2^ CFU mL^−1^ contamination levels (Figure 2(c)): significant inactivation became evident after exposure to a dose of 115.2 Jcm^−2^ [*P* = 0.009], with near complete inactivation achieved with 187.2–230.4 Jcm^−2^. Control contamination levels remained relatively unchanged [*P* = 0.255]. Significant inactivation of a 10^1^ CFU mL^−1^ seeding population was shown after a dose of 115.2 Jcm^−2^ [*P* = 0.031], with near complete inactivation achieved by exposure to doses of 201.6–230.4 Jcm^−2^ (Figure 2(d)). Control contamination levels showed no significant change compared to the exposed samples [*P* = 0.054].

#### 3.3.2. Decontamination of Plasma in a Blood Bag

**Figure 3:**
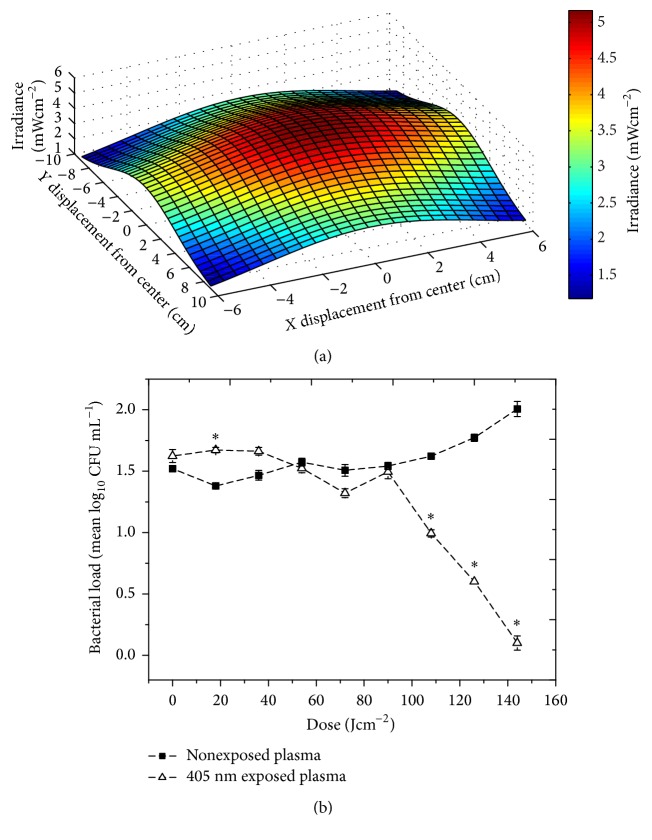
405 nm light exposure of contaminated human plasma transfusion bags. (a) Three-dimensional model demonstrating the irradiance profile across the plasma bag, with irradiance of ~5 mWcm^−2^ at the centre. (b) Inactivation of* S. aureus* contamination in 300 mL bags of human plasma by exposure to 405 nm light (*n* = 3 ± SE). Asterisks (*∗*) represent data points, where the bacterial levels in light-exposed plasma were significantly different from the equivalent nonexposed control [*P* ≤ 0.05].

**Figure 4:**
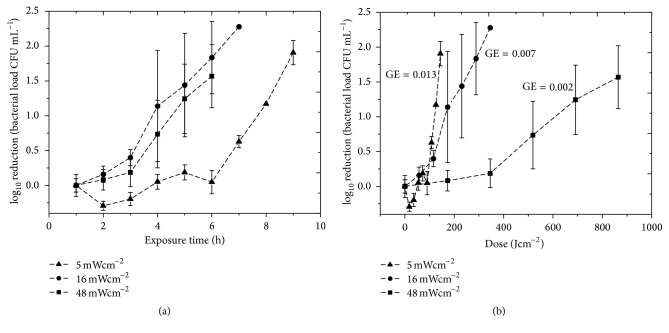
Comparison of the exposure times (a) and doses (b) required for inactivation of* S. aureus* contamination in human plasma transfusion bags. (a) Inactivation kinetics was achieved utilising irradiance of 5, 16, and 48 mWcm^−2^ at the centre of the bags. Results are presented as log_10_ reduction (CFU mL^−1^) as compared to the equivalent nonexposed control samples (*n* = 3 ± SD). Germicidal efficiency (GE) values for each irradiance are shown in (b). (GE is defined as log_10_ reduction of a bacterial population [log_10_⁡(*N*/*N*_0_)] by inactivation per unit dose in Jcm^−2^).

Inactivation of low density (10^1^–10^2^ CFU mL^−1^) bacterial contaminants within plasma transfusion bags was achieved using irradiance as low as 5 mWcm^−2^ (Figure 3(b)). A notable downward trend in contamination was observed after exposure to 108 Jcm^−2^, with a significant 0.6 log_10_ reduction in contamination [*P* ≤ 0.001]. Complete/near complete inactivation was achieved after exposure to 144 Jcm^−2^ [*P* = 0.017]. This slightly reduced inactivation rate, compared to that found within the sample dishes, is due to the lower irradiance light being used for exposure. Contamination levels in the control plasma bags rose by approximately 0.5-log_10_ [*P* = 0.052]. Similar inactivation kinetics was obtained for seeded transfusion bags exposed to irradiance of 16 and 48 mWcm^−2^, with contamination levels decreasing upon exposure to increasing treatment. Comparison of the results for the three irradiance levels used demonstrated that when looking at exposure time (Figure 4(a)) the decontamination effect observed with 16 and 48 mWcm^−2^ is relatively comparable, with inactivation being slightly slower when using the lowest irradiance of 5 mWcm^−2^. However, when looking at the actual dose levels applied (Figure 4(b)), it is apparent that the germicidal efficiency (defined as log_10_ reduction of a bacterial population [log_10_⁡(*N*/*N*_0_)] by inactivation per unit dose in Jcm^−2^ [23]) of the 5 mWcm^−2^ irradiance is greater than that of the irradiance of 16 and 48 mWcm^−2^ (*P* = 0.007 and 0.013, resp.). Comparison of exposure to doses in the region of 140–180 Jcm^−2^ highlights this difference in efficacy, with a 1.91 log_10_ reduction being achieved after exposure to 5 mWcm^−2^ for 8 h (144 Jcm^−2^), a 1.14 log_10_ reduction being achieved after exposure to 16 mWcm^−2^ for 3 h (172.8 Jcm^−2^), but only a 0.08 log_10_ reduction observed after 1 h exposure to 48 mWcm^−2^ (172.8 Jcm^−2^).

### 3.4. Optical Analysis of Plasma

**Figure 5:**
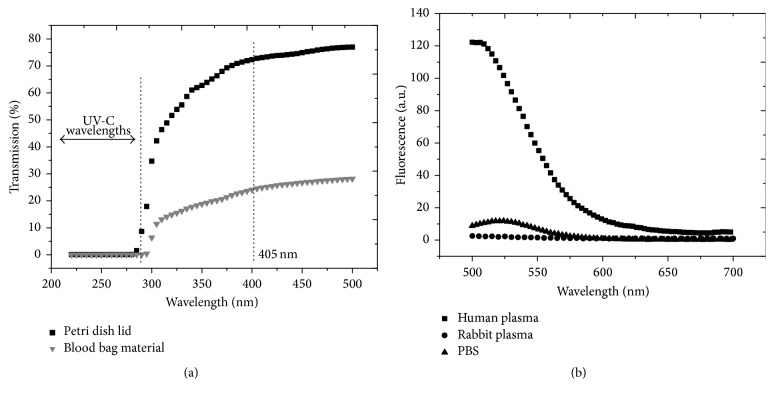
Optical analysis. (a) Transmission properties of the Petri dish and blood bag material, highlighting 405 nm and UV-C light wavelengths for reference. (b) Fluorescence emission spectra of PBS and plasma (500–700 nm), detected using an excitation wavelength of 405 nm.

Spectrophotometric analysis shows that transmission of 405 nm light through plasma is low (1-2%) compared with transparent PBS (99%), and this correlates with the longer exposure times/increased doses required for comparative microbial inactivation in plasma compared to PBS.  Figure 5(a) highlights the transmissibility of the Petri dish material and the blood bag, with results showing that 405 nm light can transmit through these materials, thus permitting decontamination of the blood plasma whilst being contained in the sample dish and blood bag. The fluorescence emission spectra of rabbit plasma and human plasma and PBS demonstrated that excitation of the suspensions at 405 nm produced no prominent fluorescence emission peaks between 500 and 700 nm (Figure 5(b)).

## 4. Discussion

In order to assess the potential of 405 nm light for decontamination of blood plasma, the penetrability and antimicrobial efficacy of 405 nm light in plasma required evaluation, and the aim of this study was to determine the antibacterial effects of 405 nm light at varying irradiance on bacteria seeded in blood plasma ranging from small volume samples up to prebagged plasma.

Initial investigation of the inactivation of bacterial contaminants in low volume (3 mL) plasma samples using 100 mWcm^−2^ 405 nm light demonstrated that successful inactivation could be achieved in both rabbit plasma and human plasma. Significantly greater doses were required for inactivation of bacterial contaminants when being suspended in plasma compared to PBS, and this is accredited to the differing optical properties of these suspending media. The opacity, and consequent low transmissibility of plasma (Figure 5(a)), reduces photon penetration through the suspension, resulting in the requirement for greater doses, compared with suspension in clear, transparent liquids such as PBS. Despite this, these proof-of-principle results demonstrate that significant inactivation of bacterial contaminants in human plasma can be achieved; and the higher the irradiance of light applied, the shorter the exposure time required for successful inactivation.

Despite the optical transmission properties of rabbit plasma and human plasma being relatively similar, slight differences were recorded between the susceptibilities of the bacterial contaminants when seeded in these media. This is likely due to the batch-to-batch variation in color and opacity of the rabbit plasma and, in particular, the human plasma. Indeed, optical analysis of a number of human plasma bag samples (*n* = 30) demonstrated variation in transmission at 405 nm between 0.2 and 25% (Maclean, Anderson, MacGregor, and Atreya; unpublished data). This is likely the reason for the large standard deviation in some of the data points in the inactivation kinetics for the prebagged plasma.

The bacterial species used in this study were selected to represent significant contaminants associated with blood components [3]. Although only three organisms were utilised, the wide antimicrobial efficacy of 405 nm light has been reported in a number of publications [20, 22, 23, 25, 36]. It is therefore expected that these organisms would also be successfully inactivated by 405 nm light when suspended in plasma. Typically, Gram-positive bacteria tend to have greater susceptibility to 405 nm light than Gram-negative bacteria [23], and this is consistent with the results reported here, with approximately 4 times greater dose required to inactivate* E. coli* in plasma, compared to the staphylococci. Interestingly, the difference between the susceptibilities of the staphylococci and* E. coli* was less pronounced when suspended in plasma compared to in PBS (4 versus 7.5 times the dose required).

The initial exposure tests in this study to establish proof of principle utilised low volumes of plasma seeded with high population densities of bacterial contaminants at a level of 10^5^ CFU mL^−1^. A more realistic scenario involves larger volumes of plasma contaminated with low microbial densities. Indeed, it has been reported that the levels of naturally occurring bacterial contamination in plasma are likely to be as low as 10–100 bacterial cells per product at the beginning of storage [37]. Accordingly, experiments were scaled up 10-fold and 100-fold using larger plasma volumes seeded with bacterial contamination levels down to 10^1^ CFU mL^−1^, using* S. aureus* as the model organism. Results demonstrated that bacterial contamination levels, even less than 10 CFU mL^−1^, can be significantly reduced in larger volumes of plasma by exposure to 405 nm light. It was interesting to note that when using similar irradiance values the bacterial inactivation rates in the 30 mL and 300 mL samples were very similar (~1.5 log_10_ reductions with a dose of 144 Jcm^−2^—Figures 2(c) and 3(b)) despite the 10-fold difference in sample volume. Although the sample volumes were different, the depths of plasma were similar (~1-2 cm in both cases), thus indicating that when using similar irradiance values it is the depth of plasma that is likely to influence the light inactivation efficacy rather than the overall sample volume. Also, results demonstrated that use of lower irradiance is likely to be more efficient, in terms of both optical energy and antimicrobial activity, compared to higher irradiance. This is possibly due to the fact that there is a critical level of photons that can be involved in the photoexcitation of the bacterial porphyrin molecules, and above this irradiance level, there is provision of excess photons which, although exposing the cells, are unable to contribute to the reaction due to the fact that there is a limit on the free porphyrin to photon ratio.

In addition to demonstrating efficacy when applied to larger volumes of plasma, these experiments highlighted that the 405 nm light disinfection effect can be achieved through transparent packaging. A similar effect was reported in a recent study which highlighted the ability of 405 nm light to decontaminate biofilms on the underside of transparent materials [38]. The ability of 405 nm light to transmit through the PVC bag layer to treat the plasma is particularly advantageous as it opens up the possibility for prebagged plasma to be treated immediately prior to storage, without the need for addition of photosensitizers, and/or passing the plasma through external decontamination systems, which can potentially introduce new contamination into the plasma products [6]. The transmissibility of 405 nm light is also a significant advantage over UV-C light, which is blocked by the PVC bag material (Figure 5(a)). Measurements in the present study demonstrated that transmission of 405 nm light through the blood component bag material resulted in an approximate 20% loss in irradiance; however, light irradiance can be increased through the use of higher power light sources in order to compensate for this loss if required. Future developments would also look to improve the uniformity of the light systems used to treat the plasma.

Published studies have identified microbial endogenous porphyrin molecules as the key photosensitive targets which initiate the lethal oxidative damage exerted by 405 nm and other violet light wavelengths [19, 32]. Since human blood also contains porphyrins and porphyrin derivatives, it was important to establish that inactivation by 405 nm light in our study was a result of the photoexcitation reaction within the microbial contaminants and not a consequence of excitation of any photosensitive molecules within the plasma, and this was evidenced by the absence of antimicrobial toxicity to bacterial contaminants seeded into the 405 nm light-exposed plasma. Qualitative analysis of the rabbit plasma and human plasma also detected no notable fluorescence emission peaks between 500 and 700 nm when excited at 405 nm, thus indicating no significant levels of free porphyrins or other photoexcitation sources within the plasma which might have acted as exogenous photosensitizers for the inactivation of the microbial contaminants.

The 405 nm light doses required in this study for the decontamination of blood plasma have been in the region of 158 Jcm^−2^ and above. These doses are relatively high compared to those typically required for other light-based methods, and this is due to the higher germicidal efficacy of UV light compared to 405 nm light [39], and the involvement of photosensitizing compounds such as riboflavin, methylene blue, and amotosalen also accelerates the antimicrobial effects of light, with doses as low as 6.24 JmL^−1^ being reported as sufficient for use [7, 40], significantly lower than 83 JmL^−1^ used in the present study (calculated based on the 158 Jcm^−2^ dose, transfusion bag dimensions, and volume). This benefit, however, is counterbalanced by the fact that photosensitizers are added to the blood products, and significant care must be taken to ensure that there is no toxicity to the blood components or the recipient due to the presence of residual photosensitizers [6]. Methods utilising UV-C light are currently under development and also demonstrate efficient microbial inactivation [16]. Although it does not require photosensitizers, UV-C is naturally more germicidal than 405 nm light; however, as mentioned, the limited penetrability of shortwave UV-C radiation means it is unable to decontaminate plasma packed in blood bags, as evidenced in the present study using 405 nm light (Figure 4(a)). The longer wavelength of 405 nm light also confers other benefits when compared to UV light, including reduced polymer degradation and increased human safety [41, 42].

Due to the absence of cells, solvent/detergent treatment, methylene blue and visible light, amotosalen and UV-A light, riboflavin and UV, and UV-C light are generally accepted as being suitable for plasma decontamination. This study has generated significant evidence of the efficacy of 405 nm light for decontamination of blood plasma as a model system to study injectable biological fluids. Since person-to-person variation in the activity of plasma proteins in healthy individuals is known to be significant, any loss in plasma integrity due to 405 nm light treatment is unlikely to have noticeable clinical impact. Further, since violet-blue light (405 nm) is relatively safer compared to already accepted UV light-based methods [39], its impact on plasma integrity has the potential to be reduced. Nonetheless, it is important in future studies to establish what effects are imparted onto plasma proteins when exposed to antimicrobial levels of 405 nm light relative to UV light exposure.

## 5. Conclusions

Overall, this study provides the first evidence that 405 nm light has the ability to inactivate bacterial contamination within biological fluids such as blood plasma. Significant inactivation of microbial contaminants was achieved in plasma samples of varying volumes held in different containers including prebagged plasma. The penetrability of 405 nm light and the nonrequirement for photosensitizing agents provide this antimicrobial method with unique benefits that could support its further development as a potential alternative to UV light-based systems. Further work is, however, required not only to extend the microbiological data but also to investigate the compatibility of 405 nm light with plasma components before its potential for plasma decontamination can be fully assessed. Although this study has focused on the antimicrobial effects of 405 nm light for the decontamination of plasma, it will also be of interest to establish whether bacterial reductions can be achieved in platelets stored* ex vivo* in plasma-based suspensions, which have a significantly greater risk of contamination due to the limitations of their storage conditions.

## References

[1] Burnouf T., Radosevich M. (2000). Reducing the risk of infection from plasma products: specific preventative strategies. *Blood Reviews*.

[2] Vasconcelos E., Seghatchian J. (2004). Bacterial contamination in blood components and preventative strategies: An overview. *Transfusion and Apheresis Science*.

[3] Brecher M. E., Hay S. N. (2005). Bacterial contamination of blood components. *Clinical Microbiology Reviews*.

[4] Wu Y. Y., Snyder E. L. (2003). Safety of the blood supply: role of pathogen reduction. *Blood Reviews*.

[5] Blajchman M. A., Klein H. G., Glynn S. A., Ness P. M. (2009). Research opportunities for pathogen reduction/inactivation of blood components: summary of an NHLBI workshop. *Transfusion*.

[6] Solheim B. G., Seghatchian J. (2006). Update on pathogen reduction technology for therapeutic plasma: an overview. *Transfusion and Apheresis Science*.

[7] Hornsey V. S., Drummond O., Morrison A., McMillan L., MacGregor I. R., Prowse C. V. (2009). Pathogen reduction of fresh plasma using riboflavin and ultraviolet light: effects on plasma coagulation proteins. *Transfusion*.

[8] Horowitz B., Bonomo R., Prince A. M., Chin S. N., Brotman B., Shulman R. W. (1992). Solvent/detergent-treated plasma: a virus-inactivated substitute for fresh frozen plasma. *Blood*.

[9] Hellstern P., Solheim B. G. (2011). The use of solvent/detergent treatment in pathogen reduction of plasma. *Transfusion Medicine and Hemotherapy*.

[10] Lambrecht B., Mohr H., Knuver-Hopf J., Schmitt H. (1991). Photoinactivation of viruses in human fresh plasma by phenothiazine dyes in combination with visible light. *Vox Sanguinis*.

[11] Seghatchian J., Struff W. G., Reichenberg S. (2011). Main properties of the THERAFLEX MB-plasma system for pathogen reduction. *Transfusion Medicine and Hemotherapy*.

[12] Schlenke P., Hervig T., Isola H. (2008). Photochemical treatment of plasma with amotosalen and UVA light: process validation in three European blood centers. *Transfusion*.

[13] Goodrich R. P., Edrich R. A., Li J., Seghatchian J. (2006). The Mirasol™ PRT system for pathogen reduction of platelets and plasma: an overview of current status and future trends. *Transfusion and Apheresis Science*.

[14] Ruane P. H., Edrich R., Gampp D., Keil S. D., Leonard R. L., Goodrich R. P. (2004). Photochemical inactivation of selected viruses and bacteria in platelet concentrates using riboflavin and light. *Transfusion*.

[15] Mohr H., Gravemann U., Bayer A., Müller T. H. (2009). Sterilization of platelet concentrates at production scale by irradiation with short-wave ultraviolet light. *Transfusion*.

[16] Seltsam A., Müller T. H. (2011). UVC irradiation for pathogen reduction of platelet concentrates and plasma. *Transfusion Medicine and Hemotherapy*.

[17] Seltsam A., Müller T. H. (2013). Update on the use of pathogen-reduced human plasma and platelet concentrates. *British Journal of Haematology*.

[18] Ramakrishnan P., Maclean M., MacGregor S. J., Anderson J. G., Grant M. H. (2016). Cytotoxic responses to 405 nm light exposure in mammalian and bacterial cells: involvement of reactive oxygen species. *Toxicology in Vitro*.

[19] Hamblin M. R., Viveiros J., Yang C., Ahmadi A., Ganz R. A., Tolkoff M. J. (2005). *Helicobacter pylori* accumulates photoactive porphyrins and is killed by visible light. *Antimicrobial Agents and Chemotherapy*.

[20] Guffey J. S., Wilborn J. (2006). *In vitro* bactericidal effects of 405-nm and 470-nm blue light. *Photomedicine and Laser Surgery*.

[21] Maclean M., MacGregor S. J., Anderson J. G., Woolsey G. A. (2008). High-intensity narrow-spectrum light inactivation and wavelength sensitivity of *Staphylococcus aureus*. *FEMS Microbiology Letters*.

[22] Enwemeka C. S., Williams D., Hollosi S., Yens D., Enwemeka S. K. (2008). Visible 405 nm SLD light photo-destroys methicillin-resistant *Staphylococcus aureus* (MRSA) in vitro. *Lasers in Surgery and Medicine*.

[23] Maclean M., MacGregor S. J., Anderson J. G., Woolsey G. (2009). Inactivation of bacterial pathogens following exposure to light from a 405-nanometer light-emitting diode array. *Applied and Environmental Microbiology*.

[24] Murdoch L. E., MacLean M., MacGregor S. J., Anderson J. G. (2010). Inactivation of *Campylobacter jejuni* by exposure to high-intensity 405-nm visible light. *Foodborne Pathogens and Disease*.

[25] Murdoch L. E., Maclean M., Endarko E., MacGregor S. J., Anderson J. G. (2012). Bactericidal effects of 405 nm light exposure demonstrated by inactivation of *Escherichia, Salmonella, Shigella, Listeria*, and *Mycobacterium* species in liquid suspensions and on exposed surfaces. *The Scientific World Journal*.

[26] Endarko E., Maclean M., Timoshkin I. V., MacGregor S. J., Anderson J. G. (2012). High-intensity 405 nm light inactivation of *Listeria monocytogenes*. *Photochemistry and Photobiology*.

[27] Wasson C. J., Zourelias J. L., Aardsma N. A. (2012). Inhibitory effects of 405 nm irradiation on Chlamydia trachomatis growth and characterization of the ensuing inflammatory response in HeLa cells. *BMC Microbiology*.

[28] MacLean M., Murdoch L. E., MacGregor S. J., Anderson J. G. (2013). Sporicidal effects of high-intensity 405 nm visible light on endospore-forming bacteria. *Photochemistry and Photobiology*.

[29] Maclean M., MacGregor S. J., Anderson J. G. (2010). Environmental decontamination of a hospital isolation room using high-intensity narrow-spectrum light. *Journal of Hospital Infection*.

[30] Bache S. E., MacLean M., MacGregor S. J. (2012). Clinical studies of the High-Intensity Narrow-Spectrum light Environmental Decontamination System (HINS-light EDS), for continuous disinfection in the burn unit inpatient and outpatient settings. *Burns*.

[31] Maclean M., Booth M., Anderson J. (2013). Continuous decontamination of an intensive care isolation room during patient occupancy using 405 nm light technology. *Journal of Infection Prevention*.

[32] Dai T., Gupta A., Huang Y.-Y. (2013). Blue light rescues mice from potentially fatal pseudomonas aeruginosa burn infection: efficacy, safety, and mechanism of action. *Antimicrobial Agents and Chemotherapy*.

[33] McDonald R., MacGregor S. J., Anderson J. G., MacLean M., Grant M. H. (2011). Effect of 405-nm high-intensity narrow-spectrum light on fibroblast-populated collagen lattices: an *in vitro* model of wound healing. *Journal of Biomedical Optics*.

[34] McDonald R. S., Gupta S., Maclean M. (2012). 405 nm light exposure of osteoblasts and inactivation of bacterial isolates from arthroplasty patients: potential for new disinfection applications?. *European Cells and Materials*.

[35] Atreya C. D., Maclean M., Anderson J. G., MacGregor S. J. Inactivation of pathogens in ex vivo blood products in storage bags using visible light.

[36] Murdoch L. E., McKenzie K., Maclean M., MacGregor S. J., Anderson J. G. (2013). Lethal effects of high-intensity violet 405-nm light on *Saccharomyces cerevisiae*, *Candida albicans*, and on dormant and germinating spores of *Aspergillus niger*. *Fungal Biology*.

[37] Brecher M. E., Holland P. V., Pineda A. A., Tegtmeier G. E., Yomtovian R. (2000). Growth of bacteria in inoculated platelets: implications for bacteria detection and the extension of platelet storage. *Transfusion*.

[38] McKenzie K., Maclean M., Timoshkin I. V., Endarko E., Macgregor S. J., Anderson J. G. (2013). Photoinactivation of bacteria attached to glass and acrylic surfaces by 405 nm light: potential application for biofilm decontamination. *Photochemistry and Photobiology*.

[39] Yin R., Dai T., Avci P. (2013). Light based anti-infectives: ultraviolet C irradiation, photodynamic therapy, blue light, and beyond. *Current Opinion in Pharmacology*.

[40] Bihm D. J., Ettinger A., Buytaert-Hoefen K. A. (2010). Characterization of plasma protein activity in riboflavin and UV light-treated fresh frozen plasma during 2 years of storage at −30°C. *Vox Sanguinis*.

[41] Andrady A. L., Hamid S. H., Hu X., Torikai A. (1998). Effects of increased solar ultraviolet radiation on materials. *Journal of Photochemistry and Photobiology B: Biology*.

[42] Matsumura Y., Ananthaswamy H. N. (2004). Toxic effects of ultraviolet radiation on the skin. *Toxicology and Applied Pharmacology*.

